# A simple, flexible and efficient PCR-fusion/Gateway cloning procedure for gene fusion, site-directed mutagenesis, short sequence insertion and domain deletions and swaps

**DOI:** 10.1186/1746-4811-5-14

**Published:** 2009-10-28

**Authors:** Ivan I Atanassov, Ilian I Atanassov, J Peter Etchells, Simon R Turner

**Affiliations:** 1 Faculty of Life Sciences, The University of Manchester, Oxford Road, Manchester, UK, M13 9PT

## Abstract

**Background:**

The progress and completion of various plant genome sequencing projects has paved the way for diverse functional genomic studies that involve cloning, modification and subsequent expression of target genes. This requires flexible and efficient procedures for generating binary vectors containing: gene fusions, variants from site-directed mutagenesis, addition of protein tags together with domain swaps and deletions. Furthermore, efficient cloning procedures, ideally high throughput, are essential for pyramiding of multiple gene constructs.

**Results:**

Here, we present a simple, flexible and efficient PCR-fusion/Gateway cloning procedure for construction of binary vectors for a range of gene fusions or variants with single or multiple nucleotide substitutions, short sequence insertions, domain deletions and swaps. Results from selected applications of the procedure which include ORF fusion, introduction of Cys>Ser mutations, insertion of StrepII tag sequence and domain swaps for *Arabidopsis *secondary cell wall *AtCesA *genes are demonstrated.

**Conclusion:**

The PCR-fusion/Gateway cloning procedure described provides an elegant, simple and efficient solution for a wide range of diverse and complicated cloning tasks. Through streamlined cloning of sets of gene fusions and modification variants into binary vectors for systematic functional studies of gene families, our method allows for efficient utilization of the growing sequence and expression data.

## Background

The rapid increase in the quantity of publicly available genome sequence information and expression data for various plant species provides an excellent resource for functional genomic studies. Such studies require a variety of cloning, modification and expression experiments [1]. Utilisation of sequences of known orthologous and/or paralogous members of gene families combined with expression data and biochemical analysis respresents a powerful resource. However, efficient utilisation of this resource requires a flexible and efficient cloning procedure. This procedure must be suited to gene and promoter cloning, site-directed mutagenesis, domain deletion and swapping, protein tagging and reporter gene fusion and must enable efficient introduction of desired DNA modification(s) and assembly of DNA fragments regardless of sequence. The recombinant DNA fragment must then be subsequently (ideally directly) cloned into *Agrobacterium *binary vectors widely used for plant transformation. Binary vectors used for *Agrobacterium *mediated plant transformation are large, vary in structure, origin of replications and tend to be cumbersome for high throughput cloning using classical techniques. A range of improvements of commonly employed procedures and cloning methods have been reported. These include expanding efficiency of classical cloning protocols which make use of restriction enzyme and ligase reactions by the use of improved binary vectors and rare cutters [2], employing site-specific recombinational DNA cloning systems [3] and application of seamless DNA fusion approaches [4].

Recombinational cloning which utilizes site-specific DNA recombination offers efficient single step transfer of DNA segments between vectors. Three recombination systems are available that allow for efficient DNA cloning: Gateway [5,6], Univector [7] and Creator [8]. The Gateway cloning system has become increasingly popular for construction of binary vectors as several sets of Gateway compatible binary vectors have become available [9-13]. These can be used for a wide range of studies including over- and inducible- gene expression, protein tagging, reporter gene fusions and gene silencing. Large numbers of entry clones generated from different plant species as well as publicly available collections [1,14] have widened the use of Gateway. More recently, Multisite Gateway recombination systems have allowed simultaneous assembly and cloning of two or three DNA fragments into destination vectors [15,16]. The Gateway cloning procedure utilizes BP and LR clonase reactions (for detailed description of the Gateway cloning system and nomenclature see [6]). The BP clonase reaction induces recombination between *attB *sites which flank the DNA fragment of interest, and *attP *sites that flank a CmR-ccdB expression cassette in a donor vector. Insertion of a DNA fragment into a donor vector using the BP clonase generates an entry clone where the inserted DNA is flanked by *attL *sites. A subsequent cloning LR step involves recombination between *attL *sites on the entry clone and *attR *sites which flank a CmR-ccdB cassette in a destination vector. As a result, a DNA fragment from an entry clone can be transferred to a destination vector (e.g. binary plant transformation vector) to produce an expression clone. In expression clones constructed in this manner the inserted DNA fragment is flanked by 25 bp *attB *sites. This is one drawback of Gateway cloning, particularly when applied to in frame gene fusion or tagging. A recent report by Dubin and co-workers [17] combines Gateway and classical cloning to eliminate insertion of *attB *sequence from the DNA fusion site, but still requires two different steps. A further drawback of Gateway is that there is no streamlined application for performing of site-directed mutagenesis, domain deletion or swapping.

Seamless DNA cloning, where two or more DNA fragments are fused with no unwanted nucleotide sequence at junction sites [4] is an alternative to recombinational cloning when precise assembly of the DNA fragments is required. Seamless cloning procedures also offer flexibility for introduction of various modifications in DNA fragments [4]. Overlapping PCR involves PCR amplification of selected DNA regions with complementary primers generating DNA fragments with overlapping ends [18]. Amplified DNA fragments are linked through annealing of their overlapping ends followed by PCR amplification of the entire assembled DNA fragment. The procedure allows for the introduction of a range of modifications at any point in the target DNA [18,19] in combination with joining of multiple DNA fragments [20]. Proof reading thermostable DNA polymerases have reduced the risk of errors introduced by PCR to acceptable levels so have expanded the application of overlap PCR for construction of long DNA molecules [20]. One drawback of the method is the low efficiency of direct cloning of assembled DNA fragments into large binary vectors using classical restriction/ligation cloning so the recombinant DNA fragment is generally first cloned into a small universal plasmid vector and subsequently re-cloned it into a binary vector. This procedure is particularly cumbersome if transfer of the recombinant DNA fragment to binary vectors with different selectable markers is required. The recent In-Fusion™ cloning system offers another attractive possibility for seamless cloning of a PCR fragment into linearized vector, as well as simultaneous assembly of two PCR fragments prior to cloning [21]. The main requirement for the procedure application is the joined DNA fragments to contain 15 bp homologous arms. However, the efficiency of In-Fusion™ cloning for construction of binary vectors and seamless assembly and cloning of multiple PCR fragments remains to be evaluated.

Arabidopsis secondary cell wall biosynthesis requires three closely related cellulose synthases *AtCesA4, AtCesA7 *and *AtCesA8 *all of which are necessary for assembly of a functional cellulose synthase complex. Questions remain as to which amino acid residues and protein domains are involved in CESA interactions, subcellular trafficking and function of the complex [22,23]. The CESA proteins offer the possibility of the kind of genomic study described above. *AtCesA *cDNAs are relatively large in size (> 3 kb) and are cumbersome for introducing DNA modifications during cloning, however we have constructed a range of CesA gene variants with nucleotide substitutions, gene fusions, domain deletions and swaps using a newly developed PCR-fusion/Gateway cloning procedure. It combines the seamless DNA fusion feature of the overlap PCR approach with the high efficiency for cloning of PCR fragments of the Getaway cloning system. Using these variants, we demonstrate that this method provides for versatile and efficient introduction of various DNA modifications followed by streamlined cloning of the modified DNA fragments directly into binary vectors.

## Methods

### Reagents and enzymes

PCR-fusion was carried out using Phusion DNA polymerase (Finnzymes; Finland) and a standard thermal cycler. Gateway recombination reactions were performed with BP Clonase II and LR Clonase II enzyme mixes (Invitrogen). Competent *E. coli *DH5α cells, were prepared according to [24]. Plasmid DNA and PCR fragments were purified using QIAprep^® ^Spin Miniprep Kit and QIAquick^® ^Gel Extraction and PCR purification kits (Qiagen, Germany). Gateway donor vector pDONR/Zeo was purchased from Invitrogen. The examples described utilize entry clones pZ1, pZ3 and pZ5 which carry *AtCesA8, AtCesA4 *and *AtCesA7 *cDNAs in pDONR/Zeo [23], respectively. The destination vector p3KC was derived from pCB2300 following the insertion of [prom *AtCesA7*/(frameA: ccd, Cm^R^)/NOS] cassette that includes a 1.7 kbp promoter sequence from *AtCesA7 *gene fused with frame A (*attR1/ccdB-CmR/attR2*) cassette (Invitrogen) and NOS terminator region, [23].

### PCR-fusion: PCR amplification and overlap extension

DNA template(s) and PCR primers are provided in figure legends. PCR-fusion involves two or three parallel PCR amplifications from plasmid template(s). PCR fusion of the amplified fragments through a single overlap extension was carried out on gel purified PCR fragments from these parallel reactions. Cycling parameters were identical for all PCR amplifications in this manuscript using reaction mix and conditions according to Phusion DNA polymerase guidelines [25]. Annealing temperatures from plasmid templates were 55°C.

For fusion of two PCR fragments we used 30 μl overlap extension reactions which contained: 16 μl mixture of the two PCR fragments (normally 8 μl for each one; approx. 200-800 ng, DNA), 6 μl of 5× Phusion HF Buffer, 3 μl of 2 mM dNTP mix, 0.3 μl of Phusion™ DNA Polymerase (2 U/μl). No primers were added to the overlap extension mixture. When three DNA fragments were fused, an 18 μl mixture of the PCR fragments (normally 6 μl for each one) was used. Generally we used equal volumes of purified PCR fragments without checking exact DNA concentrations. If the molar ratios of the amplified PCR fragments appeared to differ substantially (e.g. by more than 5-7 fold, following estimation of DNA band intensities after agarose electrophoresis), volumes from purified PCR fragments were adjusted accordingly. The reaction mix was incubated at 98°C for 30 sec., 60°C for 1 min and 72°C for 7 min. DNA obtained after the overlap extension reaction was purified using a PCR purification kit.

### Gateway cloning into Destination vector

Fused PCR fragments were recombined into a Destination vector using Gateway LR Clonase II enzyme mix kit. LR reaction mixture of 10 μl contained: 4-7 μl (approx. 50-300 ng) of DNAs purified following overlap extension, 1 μl (approx. 150 ng) of destination vector and 2 μl of Gateway LR Clonase II enzyme mix. Following 2-4 hours incubation at 25°C half of the reaction was removed, incubated with 1 μl Proteinase K solution for 10 min at 37°C and used for transformation of *E. coli *cells which were transformed as described in [24]. The remaining half of the LR reaction was incubated overnight at 25°C and subsequently used in *E. coli *transformations if the first transformation failed. In the vast majority of cases, the first *E. coli *transformation resulted in positive clones and subsequent transformations with the remaining half of the LR reaction were not required. If improved efficiency is required commercially available competent cells could be used. In the vast majority of cases, one or two of the obtained colonies were PCR checked prior to identification of a positive expression clone. The inserted DNA fragment obtained from each cloning experiment was full length sequenced using primers matching the vector regions flanking the inserted DNA fragment and gene specific primers. DNA sequencing was carried out at the sequencing facility of University of Manchester.

## Results and Discussion

### PCR-fusion/Gateway cloning procedure

#### Gene fusion

**Figure 1:**
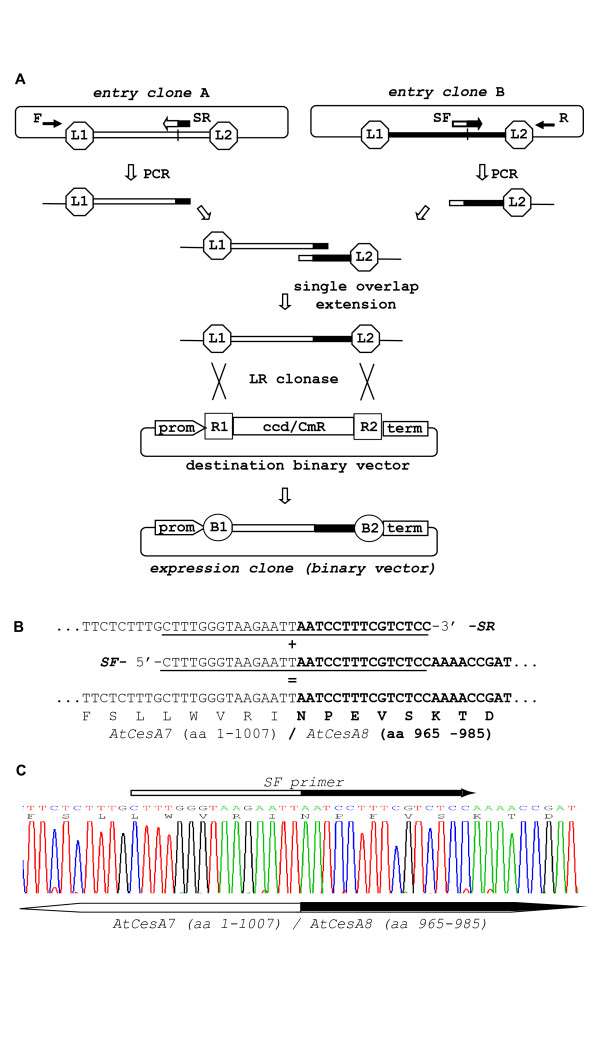
**PCR-fusion/Gateway procedure: 'gene fusion' protocol**. (**A**) Schematic of gene fusion protocol. PCR amplification from entry clones (primer pairs F × SR and SF × R) of fragments with short overlapping ends; joining of the PCR fragments by single overlap extension; direct LR clonase II mediated cloning of the assembled DNA fragment into a binary destination vector and generation of binary expression vector (expression clone). The recombination of *att*L1 and L2 sites with *att*R1 and R2 sites to give *att*B sites flanking the fused DNA fragments during the Gateway cloning are designated. Cassette enables efficient selection of entry and expression clones and contains *ccdB *and chloramphenicol resistance (CmR) genes (indicated with ccd/CmR). (**B**) Generation of *AtCesA7 *(aa: 1-1007)/*AtCesA8 *(aa: 965-985) gene fusion. Two fragments were amplified from the starting entry clones with primers: clone pZ3 (*AtCesA7*): F(M13for)-*gtaaaacgacggccagt *× SR- *ggagacgaaaggattaattcttacccaaag *and clone pZ1(*AtCesA8*): SF-*ctttgggtaagaattaatcctttcgtctcc *× R(M13rev)-*caggaaacagctatgac*. Note that primers F and R are universal M13-forward and M13-reverse primers matching the vector sequence outside the *att*L1/*att*L2 region. The sequences of the overlapping ends of the two PCR fragments and gene fusion site are presented. The primer sequences are underline. The *AtCesA8 *sequences are in bold type. (**C**) Sequencing of the DNA fusion site in binary expression vector p3K3C1 which contained a *AtCesA7 *(aa: 1-1007)/*AtCesA8 *(aa: 965-985) fusion cloned into p3KC binary destination vector. The fusion site and position of SF primer are designated on the DNA sequence.

The PCR-fusion/Gateway cloning procedure combines the advantages of overlap extension mediated seamless DNA fusion with the high cloning efficiency of the Gateway system. A PCR-fusion step involves the assembly of two or more PCR amplified DNA fragments into a fusion fragment flanked by *attL1*/*attL2 *sites. The assembled DNA fragment is subsequently cloned directly into a Gateway destination vector using an LR Clonase recombination reaction. This procedure offers exceptional flexibility for performing various gene fusion and modification applications which requires the generation of considerably fewer Gateway donor vectors than would otherwise be required. An essential requirement for the PCR-fusion/Gateway procedure is that DNA fragment(s) to be brought together in a DNA fusion reaction must be present in a Gateway donor vector (ca. Gateway entry clones; Fig. 1). In this manuscript, entry clones were made by PCR mediated addition of *attB1*/*attB2 *sites to the DNA fragment of interest which was cloned into pDONR221 or pDONR/Zeo vectors using BP Clonase reactions. PCR-fusion was then carried out according to [18], except that the standard PCR amplification step for generation of DNA fragments with overlapping ends was followed by a single extension reaction for fusion of these fragments. In most of the experiments we used 30 bp complementary PCR primers SF and SR (Fig. 1a) that matched the ends of the two DNA fragments to be fused. The application of shorter 24-28 base complementary primers was successful in few cloning experiments, as well. The 3'- half of the primers matched the 5'-end of one DNA fragment of interest. The 5'-half of the primer corresponded to the 3'-end of the DNA fragment to be fused. The other primers used for the PCR amplifications from the entry clones were universal primers matching the donor vector sequences outside of the *attL1*/*attL2 *regions. We routinely used M13 Forward and Reverse universal primers which are located outside of the *attL1*/*attL2 *region in pDONR221 or pDONR/Zeo vectors. The PCR amplifications from entry clones with specific and universal primer pairs resulted in PCR fragments which contained the corresponding *attL *site on one end and 30 bp overlapping region at the other. PCR fragments were gel-purified and used for the DNA extension mix. Following a single DNA denaturing/annealing/extension cycle, the two PCR fragments were seamlessly joined to generate a DNA fusion fragment surrounded by *attL1*/*attL2 *sites. This fragment was directly cloned into the desired destination vector using an LR Clonase recombination reaction (Fig. 1A). The described 'gene fusion' protocol offers a seamless Gateway cloning alternative to the Multisite Gateway recombination system. In addition, application of this protocol avoids the dedicated construction of specific Gateway entry clones required for Multisite Gateway [15,16].

Sequence analysis of one such 'gene fusion' experiment for construction of a chimeric *AtCesA7/AtCesA8 *gene is demonstrated (Fig. 1b, c). The C-terminus of all CESA proteins possesses a stretch of six transmembrane domains followed by a short cytoplasmic region. To elucidate the functional significance of this C-terminus region, a large part of the *AtCesA7 *cDNA including the six transmembrane region (aa: 1-1007) was seamlessly fused to the small part of *AtCesA8 *cDNA corresponding to the cytosolic C-terminus region (aa: 965-985; Fig. 1b, c).

#### Domain deletion

**Figure 2:**
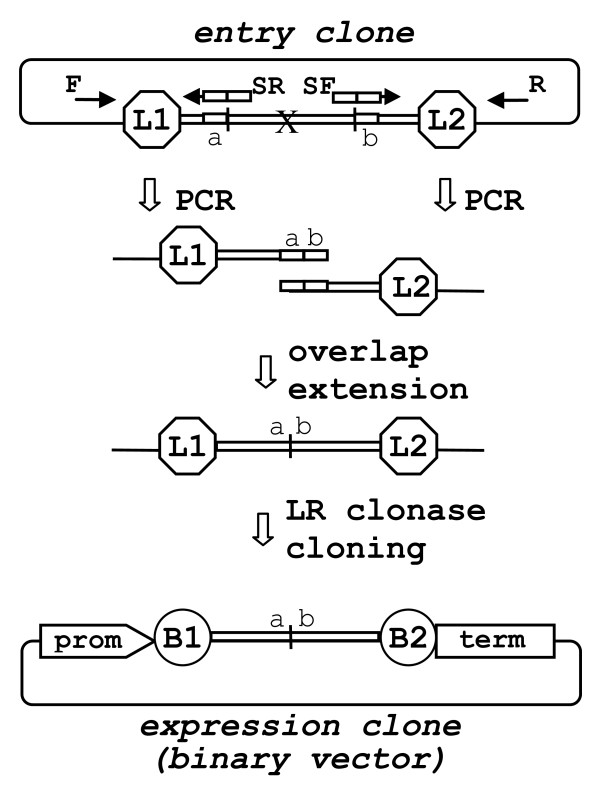
**DNA 'domain deletion' protocol**. Schematic representation of a 'domain deletion' using PCR amplification of the two terminal regions of the entry clone adjacent to the DNA domain to be deleted (designated by 'X'). F and R - represent M13 universal primers. SF and SR are complementary PCR primers consisting of two fused sections (*a *and *b*) that match regions flanking the deleted domain. The overlap extension results in assembly of the two terminal regions with overlapping ends. The new fragment with deleted DNA domain is directly LR clonase cloned into a binary destination vector producing binary expression vector, (for details see Fig. 1).

The generation and expression of a set of promoter or coding region deletion variants are an important part of many functional studies on promoter or protein domain structure. Precise DNA domain deletion is readily achieved using a modified PCR-fusion/Gateway procedure similar to the 'gene fusion' protocol described above. PCR amplification of the two terminal regions of the entry clone using PCR primers that match the flanking DNA of the region to be deleted (Fig. 2) followed by an overlap extension step produces a DNA fusion fragment in which the target DNA domain is seamlessly deleted. The protocol is likely to be useful for deletions of putative transcription factor binding sites from promoter regions or precise deletions of selected parts from coding regions. Using this method we have generated a set of expression clones carrying *Arabidopsis *CESA proteins with specific domains removed for functional analysis (data not shown).

#### Domain swap

**Figure 3:**
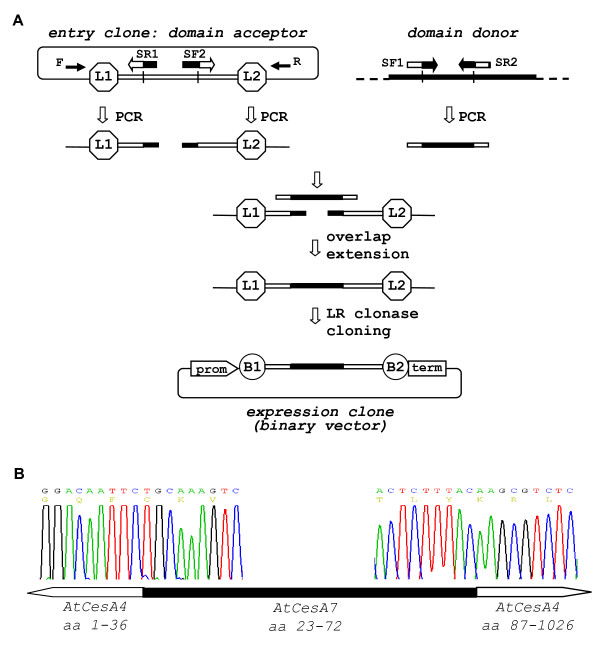
**DNA 'domain swap' protocol**. (**A**) Schematic of 'domain swap' protocol. PCR amplification from the domain acceptor entry clone (primer pairs F × SR1 and SF2 × R) of the two terminal regions flanking the DNA domain to be replaced. PCR amplification (primer pair SF1 × SR2) of the donor domain to be inserted. Overlap extension assembly of the three PCR fragments with short overlapping ends (for details see Fig. 1). Direct LR clonase cloning of the assembled fragment produces the binary expression clone. Note that SF1/SR1 and SF2/SR2 are complementary primer pairs consisting of the edges of both domain acceptor and donor. (**B**) Sequencing of the two DNA fusion sites of expression vector p3K235 that contained *AtCesA7 *(aa: 1-36)/*AtCesA4 *(aa: 23-72)/*AtCesA7 *(aa: 87-1026) domain swap variant in the p3KC binary destination vector. Cloning features: domain acceptor entry clone - pZ3 (*AtCesA7*); domain donor - plasmid cDNA clone U50150- RIKEN; primer SF1-*ctagatggacaattctgcaaagtctgtggc*; primer SF2- *tgcaacactctttacaagcgtctcagagga*; The two fusion sites at the ends of the domain swapped region are designated on the chromatograms.

Comparison of orthologous and/or paralogous members of gene families can provide important insight into the function of protein domains within gene families such as enzyme function, protein-protein interaction, protein complex assembly and sub-cellular localization and trafficking. PCR-fusion/Gateway cloning offers a streamlined protocol for fast and efficient generation of domain swap variants (Fig. 3a). The 'domain swap' protocol requires the domain acceptor gene to be preliminary cloned into an Entry vector. Donor domains were directly PCR amplified from any available DNA source in which the donor gene was present. Two pairs of specific complementary PCR primers (SF1, SR1 and SF2, SR2) that contain the sequences from donor and acceptor genes that flank the position of the swapped domain were used (Fig. 3a). Three PCR fragments were amplified. Two DNA fragments flanking the domain of interest on the acceptor gene were amplified with a gene specific and a universal vector primer from the entry clone. The third PCR fragment containing the donor domain was amplified from any available donor gene using specific primers (SF1 × SR2; Fig. 3a). Overlap extension resulted in seamless fusion of the all three PCR fragments producing the domain swap variant of the gene (Fig. 3a) which was subsequently cloned into an appropriate destination vector using an LR clonase recombination reaction. The described 'domain swap' protocol offers clear advantages over classical overlap extension method in terms of substantially increasing cloning efficiency and throughput and also over the Multisite Gateway system in terms of seamless assembly of the DNA fragments. The protocol was also successfully applied to seamless joining of more than three DNA fragments (not shown).

By using the 'domain swap' protocol, we generated a set of cellulose synthase variants in which different domains were swapped between *AtCesA4, AtCesA7 *and *AtCesA8 *genes. One CesA domain thought to be involved in protein interaction and complex assembly is a Zn-finger located close to the N-terminus [26]. A variant of *AtCesA4 *but with the Zn-finger domain from *AtCesA7 *was constructed using the 'domain swap' protocol (Fig. 3b).

#### Site-directed mutagenesis

**Figure 4:**
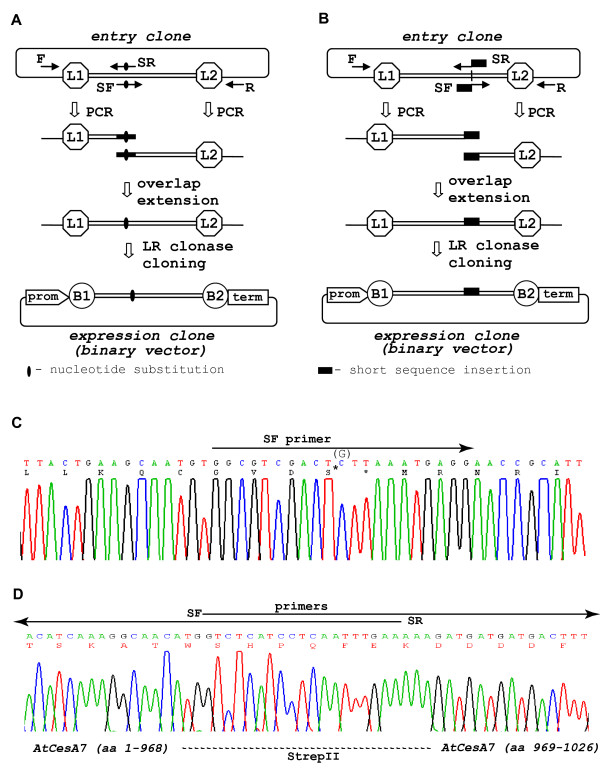
**'Site-directed mutagenesis' and 'short sequence insertion' protocols**. (**A**) Schematic 'site-directed mutagenesis' protocol. PCR amplification of the two terminal regions for creation of overlapping ends with specific primers that harbor mutated nucleotide(s). Overlap extension assembly of the PCR fragments then LR clonase cloning of the mutant fragment produces a binary expression vector. (**B**) Schematic 'short sequence insertion' protocol. PCR amplifications of two regions flanking the tag insertion position with specific PCR primers tailed with tag sequence. Overlap extension assembly of the PCR fragments with overlapped tag sequence ends, direct LR clonase cloning and generation of binary expression vector. (**C) **Sequence analysis of *AtCesA4*^*Cys*>*Ser *^cDNA mutant in which the codon of Cys^1049 ^residue was converted to Ser (GVDC^1049 ^> GVDS) using the 'site-directed mutagenesis' protocol. The mutated *AtCesA4*^*Cys*>*Ser *^cDNA is directly cloned into p3KC binary destination vector. The specific forward PCR primer (SF-*ggcgtcgactcttaaatgagg*), mutated Ser residue and Cys nucleotide from the wt cDNA clone replaced in the mutant are designated on the chromatogram. (**D**) Sequence analysis of *AtCesA7 *cDNA with *in frame *inserted StrepII tag sequence (aa: WSHPQFEK) in the middle of the spacer region between transmembrane domains 5 and 6. The specific primers tailed with StrepII sequence used for this were SF- *gtctcatcctcaatttgaaaaagatgatgatgactttggag *and SF- *ttcaaattgaggatgagaccatgttgcctttgatgtgacg*. Overlap extension assembled StrepII sequence- tailed PCR fragments are directly LR clonase cloned into p3KC binary destination vector. The inserted StrepII sequence, place of the insertion in *AtCesA7 *cDNA and the region of overlapping primers are designated on the chromatogram.

PCR-fusion/Gateway offers efficient introduction of single nucleotide mutation(s) within a gene (Fig. 4a). For 'site-directed mutagenesis' an entry clone containing the target DNA fragment is again used as the template for PCR fusion. Two specific complementary PCR primers (SF and SR; Fig. 4a) that carry single nucleotide substitution(s) at the desired positions but which otherwise match the region surrounding the nucleotide(s) to be mutated were designed. We regularly used primers with mutated nucleotide(s) flanked on both sides by 10-13 bases. PCR amplifications from the entry clone with pairs of specific and universal primers produced two PCR fragments containing the corresponding *attL *site at one end and overlapping region with mutated nucleotide(s) at the other. Following the single overlap extension step, the recombinant DNA fragment containing the mutated nucleotide(s) was directly cloned using LR Clonase into a destination vector. The protocol works well when several closely located nucleotides have to be mutated (Fig. 4c). Where mutagenesis of distantly located nucleotides was required, nucleotide substitutions were inserted simultaneously through PCR amplification and fusion of three or more DNA regions.

We extensively used the 'site-directed mutagenesis' protocol to generate sets of various nucleotide substitution variants of secondary cell wall *AtCesA *genes. We made a series of Cys>Ser mutants in which single or group of Cys residues were converted to Ser through introducing single nucleotide mutation(s) in the respective codons. Sequence analysis of a variant of *AtCesA4 *in which the last Cys was converted to Ser (GVDC^1049^> GVDS) is shown (Fig. 4c).

#### Short sequence insertions

Construction of vectors for expression of tagged proteins generally employs 'classical' cloning with restriction enzyme and ligase reactions or uses the Gateway system [12,27] which possesses the drawback of addition of *attB *sequence between the tag and coding sequence. Hybrid Gateway cloning systems [17] combine these procedures allowing better control over linker sequence but still involves less efficient restriction and ligation cloning. Furthermore, current tag-cloning procedures are well suited for fusion of tags to the N- or C- terminus of proteins, but are not applicable for insertion of tag sequence into specific locations inside the coding regions. The majority of the currently employed epitope tags have short nucleotide sequences [28] and as such are easily incorporated into primer sequences used in PCR fusion/Gateway (Fig. 4b). The protocol is similar to those above but with the 3' part of primers consisting of 12-15 bases that match the region of DNA sequence flanking the tag insertion position and a 5' tail with the nucleotide sequence of the tag. PCR with specific and 'universal' primers resulted in amplifications of two fragments of target ORF with overlapping ends containing the fused in frame tag sequence. The assembled ORF with inserted tag sequence was directly cloned into a Gateway expression vector thus allowing seamless insertion/fusion of tag sequence in any position of target ORF. The tag-ORF construct could be inserted directly in wide range of vectors for expression in bacterial, plant and other eukaryotic expression systems.

We used the 'short sequence insertion' protocol for insertion of a StrepII tag sequence internally in the *AtCesA7 *gene coding sequence. The cellulose synthase CESA plasma membrane proteins possess 8 trans-membrane domains and the rest of the protein is mainly cytosolic. So far, functional 6xHis-, FLAG- and StrepII- tagged CESA proteins were expressed only when the tag sequence was inserted in the cytosolic N- terminus of the proteins [23]. The functionality of the recombinant tag proteins with an epitope tag outside the plasma membrane was untested. This experiment required the tag sequence to be inserted in short spacer regions between the trans-membrane domains located outside the plasma membrane. The sequence analysis of one such *AtCesA7 *with a StrepII tag inserted in the short spacer region between transmembrane domains 5 and 6 is demonstrated (Fig. 4d).

#### Procedure efficiency, PCR error, construct pyramiding

The described PCR-fusion/Gateway cloning procedure is simple to plan and straightforward in application. The only differences between the different cloning protocols and experiments are the DNA templates and specific primers used. Thus the PCR-fusion/Gateway cloning could be readily scaled up using reaction master mixes and by performing multiple cloning experiments simultaneously. We successfully performed 12 to 15 cloning experiments in one run, which substantially reduced the time per cloning experiment. Such multiple cloning experiments were completed to the point of *E. coli *transformation within one to two working days. The use of the same basic PCR-fusion protocol, gel and PCR purification kits combined with a highly efficient Gateway recombination cloning step provides reproducible high efficiency of the entire cloning procedure. In the majority of cases, testing of one or two colonies from each experiment was sufficient to obtain a positive clone. If necessary, affordable high throughput application of the cloning procedure could be easily built up through automisation of gel, PCR reaction and plasmid DNA purification steps using commercially available robotic systems (e.g. 'QIAcube', Qiagen, Germany).

One limitation of overlapping PCR has been PCR errors introduced in the amplified and cloned DNA fragments. Recently developed proofreading polymerases have reduced the PCR error rate [20]. We intensively used Phusion DNA polymerase in all PCR-fusion/Gateway cloning experiments which proved to be accurate, and gave high yields of long amplicons using shorter extension times [25]. The PCR-fusion step in our cloning procedure minimizes the possibility of PCR errors as a single PCR amplification step is used instead of two successive PCR amplifications employed in the 'classical' overlap extension PCR protocol [18-20]. Since the PCR-fusion step is followed by highly efficient Gateway cloning, the amount of fused DNA fragments generated in a single overlap extension is sufficient to obtain a good number of positive clones from each cloning experiment. We performed more than 50 independent PCR-fusion/Gateway cloning experiments involving *AtCesA *genes (cDNA lenghts longer than 3 kb). Ten to more than hundred colonies were obtained from each cloning experiment. Positive clones were obtained from the all experiments and in nearly all of them the testing of one or two colonies was sufficient to identify a positive clone. The inserted DNA fragment in one positive expression clone (binary vector) from each cloning experiment was full length sequenced (> 160 kb total length of cloned and sequenced DNAs) and we found no sequence errors generated by the PCR-fusion. This suggests that the described procedure, together with the use of proofreading DNA polymerases could be successfully applied for PCR-fusion and cloning of longer DNA fragments.

**Figure 5:**
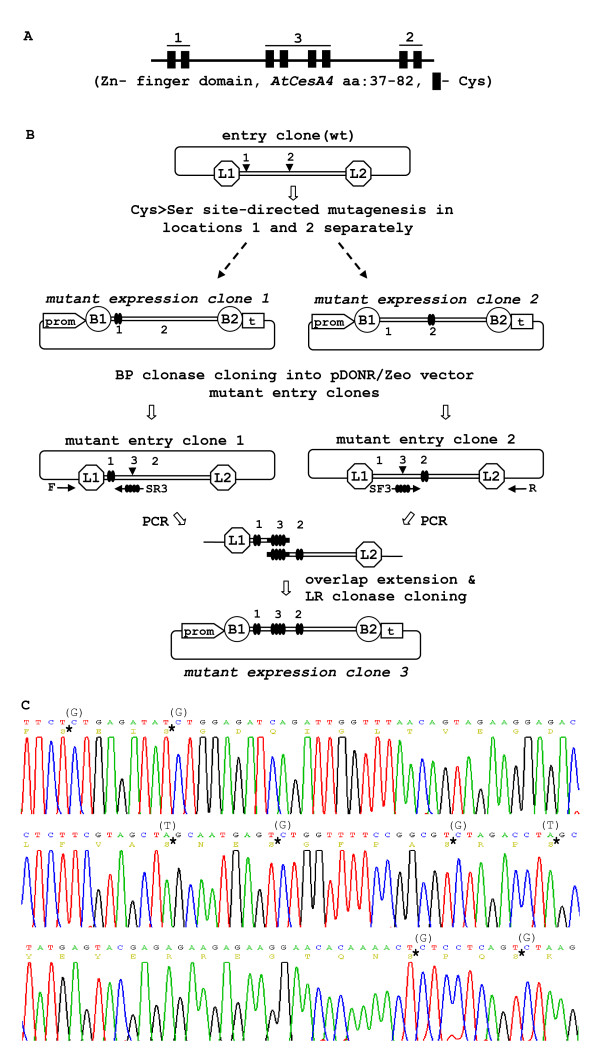
**Pyramiding of the modifications/constructs created by the PCR-fusion/Gateway procedure**. An example for pyramiding of gene modifications engineered by the PCR-fusion/Gateway procedure Showing the construction of an *AtCesA7 *cDNA mutant in which all eight Cys codons are mutated to Ser. Note that the pyramiding scheme described could be applied for all described cloning protocols. (**A**) General Cys residue structure of the Zn-finger domain of the CESA proteins. The two pairs of Cys residues which are mutated separately are designated. (**B**) Schematic of pyramiding of the Cys-residue mutagenesis in the Zn-finger domain of *AtCesA7*. Step 1: two *AtCesA7*^*Cys*>*Ser *^cDNA mutants are constructed in parallel, starting from wt *AtCesA7 *cDNA using the 'site-directed mutagenesis' protocol (Fig. 4a). In mutant expression clone 1 the codons of first pair of Cys are converted to Ser and in clone 2 the last pair. The mutated cDNA from expression clones 1 and 2 are generated using BP clonase and Gateway donor vector (pDONR/Zeo) to obtain mutant entry clones 1 and 2. Terminal regions from each of the mutated entry clones are PCR amplified with specific primers containing mutated codons of the remaining internal Cys residues of the Zn-finger. Overlap extension and cloning of the assembled DNA fragment into p3KC binary destination vector resulted in expression clone 3 in which all eight Cys codons are mutated to Ser. (C) Sequence analysis of the region of the Zn-finger domain mutant in expression clone 3. The converted Cys>Ser residues are marked with (*) and replaced nucleotides are shown in brackets.

Stepwise introduction of modifications into target DNA and pyramiding of the constructed expression vectors through transfer of modified DNA fragments between clones is essential for a wide variety of experiments. Gateway cloning allows a modified DNA fragment from an expression clone to be back-cloned into an Entry vector using BP Clonase [6] and as such allows flexibility for pyramiding introduced modifications in the starting DNA fragment. By re-cloning modified DNA from an expression clone to generate a modified entry clone followed by a second forward modification we generated successive site-directed mutagenesis of the Cys residues from the Zn-finger domain of the *AtCesA7 *gene using the PCR-fusion/Gateway cloning procedure (Fig 5). The amino-terminal of all CESA cellulose synthases possess 8 (4 × 2) cysteine residues that form a RING-type zinc finger domain (Fig. 5a) [22]. To evaluate the functional significance of the cysteines at different positions in the domain, we generated three different AtCesA7^Cys>Ser ^mutants. In the first round, two Cys>Ser clones were generated independently using the 'site-directed mutagenesis' protocol. In clone 1, the first two Zn-finger cysteines were converted to Ser. In clone 2 the last pair of Zn-finger cysteines were mutated to Ser (Fig. 5b). These two Cys>Ser clones were back-cloned into the pDONR/Zeo vector using a BP clonase recombination reaction producing entry clones 1 and 2 (Fig. 5b). The two mutated entry clones were subsequently used in a second round of 'site-directed mutagenesis' cloning, which resulted in an expression clone with all eight *Cys *codons converted to *Ser *(Fig. 5c). The pyramiding of constructs could involve any of the cloning options described above for PCR-fusion/Gateway.

#### Procedure requirements

The only prerequisites for application of the PCR-fusion/Gateway cloning procedure are the availability of entry clones containing manipulated DNA fragments and suitable destination binary vectors for cloning of the assembled DNA fragment. The easy *de novo *construction of entry clones and destination vectors [6], as well as publicly-available large entry clone collections [14] and sets of diverse destination vectors [10,12,13] provide a excellent background for the application of the described procedure.

## Conclusion

The PCR-fusion/Gateway cloning procedure described combines seamless DNA fusion cloning with the power of the Gateway system. The procedure offers a simple, fast and efficient method of performing gene fusions and wide range of gene modifications including site-directed mutagenesis, short sequence insertions, domain deletion and swapping, combined with direct cloning into destination binary vectors. The procedure can be applied to various scales including high throughput applications and possible automation of the main labour intensive steps. The streamlined cloning of the modified DNA fragments into new entry clones allows for flexible utilisation of binary vectors with different selectable markers that allows for pyramiding of different DNA modification.

## Competing interests

The authors declare that they have no competing interests.

## Authors' contributions

IA: conceived, designed and carried out the experiments, and prepared the manuscript. IIA: designed and carried out the experiments. PE and ST: designed the experiments and prepared the manuscript. All authors read and approved the final manuscript.

## References

[B1] Hilson P (2006). Cloned sequence repertoires for small- and large-scale biology. Trends Plant Sci.

[B2] Dafny-Yelin M, Tzfira T (2007). Delivery of multiple transgenes to plant cells. Plant Physiol.

[B3] Marsischky G, LaBaer J (2004). Many paths to many clones: a comparative look at high-throughput cloning methods. Genome Res.

[B4] Lu Q (2005). Seamless cloning and gene fusion. Trends Biotechnol.

[B5] Hartley JL, Temple GF, Brasch MA (2000). DNA cloning using in vitro site-specific recombination. Genome Res.

[B6] Invitrogen Gateway^® ^Technology Manual. http://tools.invitrogen.com/content/sfs/manuals/gatewayman.pdf.

[B7] Liu Q, Li MZ, Leibham D, Cortez D, Elledge SJ (1998). The univector plasmid-fusion system, a method for rapid construction of recombinant DNA without restriction enzymes. Curr Biol.

[B8] (2000). Creator DNA Cloning & Expression System. Clontechniques.

[B9] Karimi M, Inze D, Depicker A (2002). GATEWAY vectors for Agrobacterium-mediated plant transformation. Trends Plant Sci.

[B10] Curtis MD, Grossniklaus U (2003). A gateway cloning vector set for high-throughput functional analysis of genes in planta. Plant Physiol.

[B11] Nakagawa T, Suzuki T, Murata S, Nakamura S, Hino T, Maeo K, Tabata R, Kawai T, Tanaka K, Niwa Y (2007). Improved Gateway binary vectors: high-performance vectors for creation of fusion constructs in transgenic analysis of plants. Biosci Biotechnol Biochem.

[B12] Karimi M, Depicker A, Hilson P (2007). Recombinational cloning with plant gateway vectors. Plant Physiol.

[B13] Earley KW, Haag JR, Pontes O, Opper K, Juehne T, Song K, Pikaard CS (2006). Gateway-compatible vectors for plant functional genomics and proteomics. Plant J.

[B14] The Arabidopsis Information Resource. http://www.arabidopsis.org/.

[B15] Sasaki Y, Sone T, Yoshida S, Yahata K, Hotta J, Chesnut JD, Honda T, Imamoto F (2004). Evidence for high specificity and efficiency of multiple recombination signals in mixed DNA cloning by the Multisite Gateway system. J Biotechnol.

[B16] Karimi M, De Meyer B, Hilson P (2005). Modular cloning in plant cells. Trends Plant Sci.

[B17] Dubin MJ, Bowler C, Benvenuto G (2008). A modified Gateway cloning strategy for overexpressing tagged proteins in plants. Plant Methods.

[B18] Horton RM, Hunt HD, Ho SN, Pullen JK, Pease LR (1989). Engineering hybrid genes without the use of restriction enzymes: gene splicing by overlap extension. Gene.

[B19] Ho SN, Hunt HD, Horton RM, Pullen JK, Pease LR (1989). Site-directed mutagenesis by overlap extension using the polymerase chain reaction. Gene.

[B20] Shevchuk NA, Bryksin AV, Nusinovich YA, Cabello FC, Sutherland M, Ladisch S (2004). Construction of long DNA molecules using long PCR-based fusion of several fragments simultaneously. Nucleic Acids Res.

[B21] (2007). In-Fusion™ PCR Cloning Kits--FAQs. Clontechniques.

[B22] Taylor NG (2008). Cellulose biosynthesis and deposition in higher plants. New Phytol.

[B23] Atanassov II, Pittman JK, Turner SR (2009). Elucidating the mechanisms of assembly and subunit interaction of the cellulose synthase complex of Arabidopsis secondary cell walls. J Biol Chem.

[B24] Inoue H, Nojima H, Okayama H (1990). High efficiency transformation of Escherichia coli with plasmids. Gene.

[B25] NewEnglandBiolabs Phusion™ High-Fidelity DNA Polymerase. Manual.

[B26] Jacob-Wilk D, Kurek I, Hogan P, Delmer DP (2006). The cotton fiber zinc-binding domain of cellulose synthase A1 from Gossypium hirsutum displays rapid turnover in vitro and in vivo. Proc Natl Acad Sci USA.

[B27] Nakagawa T, Kurose T, Hino T, Tanaka K, Kawamukai M, Niwa Y, Toyooka K, Matsuoka K, Jinbo T, Kimura T (2007). Development of series of gateway binary vectors, pGWBs, for realizing efficient construction of fusion genes for plant transformation. J Biosci Bioeng.

[B28] Terpe K (2003). Overview of tag protein fusions: from molecular and biochemical fundamentals to commercial systems. Appl Microbiol Biotechnol.

